# Anatomy-driven strategies and mid-term outcomes of branched endovascular repair of the aortic arch: a single-center cohort study

**DOI:** 10.1186/s13019-026-03884-6

**Published:** 2026-02-09

**Authors:** Bin Zhao, Zongwei Liu, Hao Liang, Jiaxue Bi, Xiangchen Dai

**Affiliations:** ¹Department of Vascular Surgery, Tianjin Medical University General Hospital, Tianjin, China

**Keywords:** Aortic arch, Aortic anatomy, Thoracic endovascular aortic repair (TEVAR), Branched endograft, Single-Branch, Double-Branch, Mid-term outcomes, Age

## Abstract

**Background:**

Thoracic endovascular aortic repair (TEVAR) with branch reconstruction is an alternative to open surgery for aortic arch pathologies. However, mid-term data comparing different strategies are limited. This study aimed to evaluate the mid-term outcomes of single- and double-branch TEVAR and analyze the performance of different single-branch reconstruction techniques.

**Methods:**

We retrospectively analyzed 198 consecutive patients who underwent TEVAR for aortic arch lesions with either single-branch (*n* = 169) or double-branch (*n* = 29) reconstruction at a single center between February 2015 and December 2023. The primary endpoint was the mid-term all-cause mortality. The secondary endpoints included technical success, perioperative safety, aorta-related complications, and reintervention rates. Kaplan-Meier survival analysis, multivariable Cox regression, and Restricted Mean Survival Time (RMST) analysis were performed. The influence of age was explored using Restricted Cubic Splines (RCS), modeling age continuously.

**Results:**

The overall technical success rate was 96.0%. The mean follow-up duration was 40.5 months. There was no significant difference in mid-term all-cause mortality between the single- and double-branch groups (Log-rank *P* = 0.710). Multivariable Cox regression identified older age as an independent predictor of mortality (HR 1.047 per year, 95% CI 1.012–1.084, *P* = 0.009) but not the number of branches reconstructed (*P* = 0.611). Among the single-branch techniques, RMST analysis confirmed no significant difference in mid-term survival (all pairwise *P* > 0.050). An inverse association between age and aorta-related complications was suggested (OR 0.957, 95% CI 0.923–0.993, *P* = 0.020), which most plausibly reflects selection bias and warrants confirmation in larger, multicenter studies.

**Conclusion:**

In this single-center cohort study, single- and double-branch TEVAR yielded no statistically significant difference in mid-term survival. Age, and not the number of reconstructed branches, was the primary predictor of mortality. All single-branch techniques showed comparable mid-term survival, supporting an individualized, anatomy-driven approach. The observed inverse association between age and aorta-related complications highlights the critical role of patient selection in these complex procedures and warrants confirmation in larger, multicenter studies with standardized anatomical assessment and imaging follow-up.

## Introduction

Thoracic aortic aneurysms and acute aortic syndromes (AAS), the latter including aortic dissection, intramural hematoma, and penetrating aortic ulcer, are relatively uncommon but are associated with substantial mortality, necessitating timely management [1, 2]. Initial medical management often involves oral antihypertensive therapy for blood pressure control [3]. However, surgical intervention is frequently necessary for patients with aneurysms posing a risk of rupture or complications. Traditionally, open surgery has been the gold standard for addressing lesions of the ascending aorta and the aortic arch. These procedures often require hypothermic circulatory arrest and cardiopulmonary bypass and remain associated with non-trivial neurologic and systemic risks despite advances in perioperative management [4–6]. In contrast, endovascular aortic repair is associated with reduced perioperative morbidity and mortality. With ongoing improvements in endovascular devices and operator experience, endovascular aortic repair has evolved substantially [7]. Thoracic endovascular aortic repair (TEVAR) has emerged as the preferred surgical modality for treating various thoracic aortic pathologies including descending aortic aneurysms and Stanford type B aortic dissection [8, 9]. Consequently, the application of TEVAR for aortic arch lesions has expanded, particularly in patients with a favorable anatomy and significant comorbidities, establishing it as a primary treatment consideration in selected cases.

However, the aortic arch and its supra-aortic branch vessels are characterized by intricate anatomical configurations and complex hemodynamic profiles. Furthermore, elderly patients frequently exhibit calcified or thrombosed lesions in the aortic arch and its branches. Notably, a substantial proportion of the population demonstrates variations in aortic arch anatomy, with approximately 35% of individuals presenting with such variations [10]. These factors contribute to considerable interindividual variability in the selection and deployment of stent grafts prior to surgery. Consequently, achieving successful stent placement and ensuring long-term integrity of graft materials present significant challenges for cardiovascular surgeons. Securing an adequate proximal landing zone is crucial for stent-graft placement. When aortic arch lesions are in close proximity to the origin of the branch vessels, reconstructing the blood flow to these branches becomes imperative to ensure sufficient anchoring and maintain cerebral perfusion, thereby preventing cerebral ischemia and hypoxia.

The primary endovascular techniques currently employed for aortic arch branch reconstruction include the chimney technique, fenestration technique, branched stent-grafts, and hybrid techniques. Crucially, technique selection is anatomy-driven and depends on factors such as proximal landing zone length, branch vessel angulation, and the presence of calcification or thrombus. Although these techniques have shown favorable short-term outcomes, clinically important adverse events, including endoleaks, stent-induced new entry (SINE), and retrograde type A dissection (RTAD), remain concerns. However, comparative evidence regarding how the number of reconstructed branches and the reconstruction technique influence mid-term outcomes, including survival, complication, and reintervention rates, remains scarce. To address this gap, we conducted a retrospective analysis of patients who underwent endovascular repair of the aortic arch between 2015 and 2023. This study aimed to evaluate mid-term outcomes associated with these anatomy-driven repair strategies by comparing single- and double-branch repairs and assessing the relative effectiveness of commonly used single-branch reconstruction techniques.

## Methods

### Study design and patient population

**Fig. 1:**
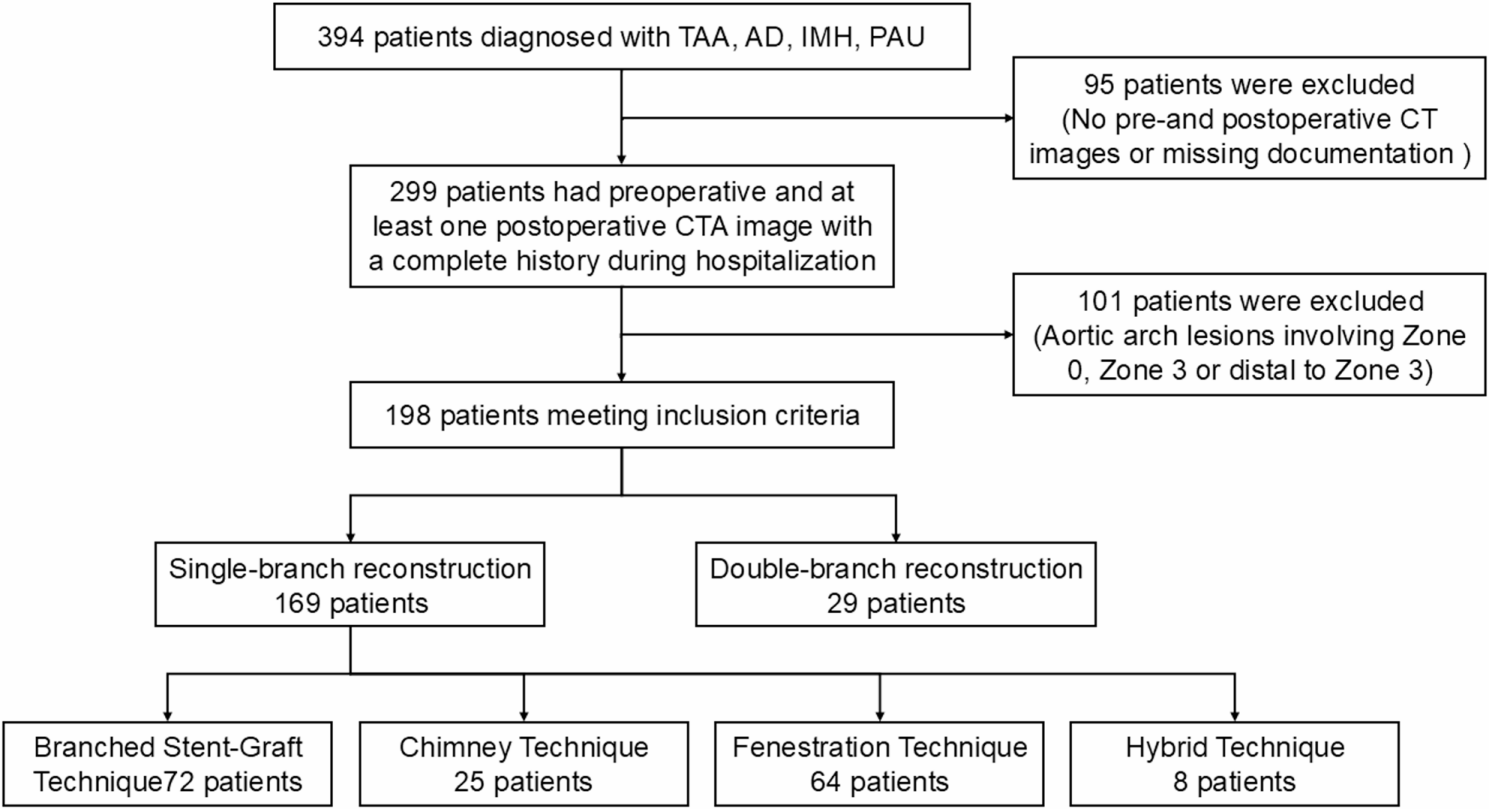
Patient Enrollment Flowchart. Diagram illustrating the process of patient screening and enrollment for the study. *CTA: Computed Tomography Angiography*

This retrospective cohort study analyzed data from patients who underwent TEVAR for aortic arch lesions at a single tertiary center between February 2015 and December 2023. The study population included patients diagnosed with thoracic aortic aneurysm, aortic dissection, intramural hematoma, or penetrating aortic ulcer involving Ishimaru Zones 1 or 2 of the aortic arch who required supra-aortic branch reconstruction. Patient enrollment is shown in Fig. 1. Briefly, 394 patients were initially assessed, and 198 met the inclusion criteria.

Key inclusion criteria were age ≥ 18 years, a definitive diagnosis requiring TEVAR with reconstruction of one or more supra-aortic branches using physician-modified branched stent grafts, chimney techniques, fenestration techniques, or hybrid procedures, and the availability of adequate imaging and medical records. Hybrid procedures were defined as surgical supra-aortic bypass combined with endovascular stent-graft implantation during the same treatment strategy. The major exclusion criteria were lesions confined to Zone 0, Zone 3, or distal to Zone 3; prior thoracic aortic surgery at the lesion site; and incomplete imaging or documentation. The study protocol was approved by the Medical Ethics Committee of Tianjin Medical University General Hospital (No: IRB2024-YX-321-01). The requirement for individual informed consent was waived because this was a retrospective study using fully anonymized data collected during routine clinical care, and the study posed minimal risk to participants.

### Endpoints and definitions

The primary endpoint of this study was mid-term all-cause mortality.

Secondary endpoints included technical success, perioperative safety outcomes including 30-day or in-hospital all-cause mortality and major adverse events, mid-term aorta-related complications, mid-term branch stent patency, and mid-term overall reintervention rates. Technical success was defined as successful device deployment with complete exclusion of the primary lesion, patency of all targeted branch vessels, and absence of type I or type III endoleak on completion angiography. Type II endoleak was documented but was not routinely classified as technical failure. However, one early-era hybrid case with persistent type II endoleak related to the left subclavian artery (LSA) despite intraoperative repeat embolization was operationally recorded as a technical failure in our dataset, and this exception is reported transparently in the Results. Emergency surgery referred to TEVAR performed within 48 h of hospital admission for acute aortic syndrome or symptomatic presentation.

Perioperative adverse events were a composite of significant complications that occurred within 30 days or during index hospitalization. These included access site issues such as thrombosis, rupture, or pseudoaneurysm requiring intervention; acute kidney injury, defined as an increase in serum creatinine by 0.5 mg/dL or more or by 50% or more from baseline; or a new need for dialysis, stroke, myocardial infarction, and lower limb or visceral embolism.

Cerebrovascular events were captured as the occurrence of stroke during the perioperative period or follow-up based on clinical documentation and available imaging reports when applicable.

Aorta-related complications were assessed throughout the perioperative and follow-up periods and included endoleak of any type, proximal stent-induced new entry (pSINE), distal stent-induced new entry (dSINE), and retrograde type A dissection (RTAD). Endoleaks requiring treatment were additionally captured as reintervention events. Because a single patient could experience more than one aorta-related complication, the sum of component events may exceed the number of patients with at least one aorta-related complication. Branch stent restenosis was defined as severe in-stent stenosis of at least 75% that required endovascular reintervention, confirmed primarily by computed tomography angiography. Branch stent occlusion was also recorded. Duplex ultrasound, when available, was used mainly to evaluate accessible cervical segments of supra-aortic branches, whereas intrathoracic segments were assessed primarily by computed tomography angiography. Reintervention was defined as a secondary procedure to treat TEVAR-related complications. Aorta-related mortality was defined as death directly due to aortic pathology or TEVAR complications.

### Procedure details

The selection of the aortic arch branch reconstruction technique (physician-modified branched stent graft, chimney, fenestration, or hybrid) and the number of branches reconstructed (single or double) were determined by a multidisciplinary team of experienced vascular surgeons. Decisions were individualized based on patient anatomy, lesion characteristics including Ishimaru landing zone classification, clinical urgency, and surgeon preference, following a thorough discussion of the risks and benefits with the patient or their representatives. The thoracic aortic main body was based on commercially available thoracic stent graft systems, primarily including Relay, Valiant, Gore, and Fabulous devices. In the physician-modified cases, fenestrations were created either on the operating table or as a preoperative modification. For preoperative modification, the basic preparation process and technical considerations have been described in our group’s previous report on physician-modified fenestrated thoracic endografts. Supra-aortic revascularization was achieved using chimney grafting, fenestration with bridging covered stents, or physician-modified branched configurations according to anatomical feasibility. In this context, antegrade refers to branch incorporation performed via femoral access, whereas retrograde refers to branch incorporation performed via upper-extremity or cervical access into the target supra-aortic artery. In eight patients, a hybrid strategy was used, consisting of carotid-to-subclavian bypass followed by intentional LSA coverage during TEVAR, thereby avoiding branch or fenestration reconstruction of the LSA. LSA occlusion was achieved predominantly by surgical ligation; in selected early cases, endovascular embolization was performed instead of ligation based on intraoperative considerations. Device sizing followed centerline computed tomography angiography measurements and manufacturer recommendations. As a general strategy, we used conservative proximal oversizing of approximately 5 to 10% for aortic dissection, and 10 to 20% for thoracic aortic aneurysm. For intramural hematoma and penetrating aortic ulcer, sizing was individualized with a conservative approach consistent with acute aortic syndrome practice, taking into account landing zone quality and aortic wall fragility [11]. 

### Data collection and Follow-up

Clinical data were retrospectively collected from electronic medical records and imaging archives. This included patient demographics, comorbidities, lesion details, procedural specifics such as techniques and devices, operative time, contrast volume, and fluoroscopy time, along with perioperative and follow-up outcomes. Postoperative surveillance typically involves computed tomography angiography (CTA) at 1, 6, and 12 months and annually thereafter or as clinically indicated. Follow-up data on mortality, reinterventions, aorta-related complications, and branch stent status were obtained from outpatient visits, imaging reports, and telephone interviews. For patients who did not return for scheduled imaging follow-up, structured telephone interviews were used to ascertain survival status and major clinical events, which was considered a limitation for imaging-defined endpoints. The mean follow-up duration for the cohort was 40.5 months.

### Statistical analysis

Statistical analysis was performed using R software (version 4.4.1, R Foundation for Statistical Computing, Vienna, Austria) and SPSS software (version 25.0, IBM Corp., Armonk, NY, USA). Continuous variables were presented as mean ± standard deviation (SD) or median (interquartile range, IQR) and compared using Student’s t-test, Mann-Whitney U test, or Kruskal-Wallis test, as appropriate. Categorical variables were presented as counts and percentages and compared using the chi-squared test or Fisher’s exact test, and time-to-event outcomes, such as survival and freedom from reintervention, were analyzed using the Kaplan-Meier method, with group comparisons made using the log-rank test. Multivariable Cox proportional hazards regression models were used to identify independent predictors of all-cause mortality, yielding hazard ratios (HR) and 95% confidence intervals (CI). The proportional hazards assumption for these models was checked using Schoenfeld residuals, and Restricted Mean Survival Time (RMST) analysis was employed for robust comparisons when this assumption was violated, with RMST differences and 95% CIs calculated at the relevant time points. Univariable logistic regression was used to explore the relationship between age and selected binary outcomes, including follow-up all-cause mortality and aorta-related complications. Analyses for branch stent restenosis requiring reintervention were descriptive because of the low event count. To flexibly model and visualize potential nonlinear associations between age and these outcomes, Restricted Cubic Splines (RCS) with 3, 4, and 5 knots were utilized in logistic regression models, presenting odds ratios (OR) and 95% CIs. Model fit and nonlinearity were assessed using likelihood ratio and Wald tests. A two-sided P-value of less than 0.05 was considered statistically significant for all analyses. Python (Version 3.13.0) was also used for specific data processing or analysis scripts.

## Results

### Patient cohort and enrollment

From February 2015 to December 2023, 394 patients diagnosed with aortic arch lesions, including thoracic aortic aneurysm, aortic dissection, intramural hematoma, or penetrating aortic ulcer, were assessed for eligibility at our center. Ninety-five patients were excluded because of inadequate pre- and postoperative CTA images or missing critical medical documentation. Of the remaining 299 patients, 101 were excluded because their lesions involved Zone 0 or Zone 3, or were distal to Zone 3, falling outside the study’s focus on Zone 1 and Zone 2 reconstructions. Consequently, 198 patients meeting all the inclusion criteria were included in this retrospective analysis, comprising 169 (85.4%) who underwent single-branch reconstruction and 29 (14.6%) who underwent double-branch reconstruction (Fig. 1).

### Comparison of Single-Branch versus Double-Branch TEVAR

Although double-branch reconstructions required longer operative times, no statistically significant differences were observed between the double-branch and single-branch groups in mid-term all-cause mortality or major complications. Baseline demographic and clinical characteristics, detailed in Table 1, were largely comparable between the groups, including mean age (single-branch: 56.3 ± 14.3 years vs. double-branch: 58.3 ± 13.8 years, *P* = 0.481), sex distribution, and most comorbidities. However, significant differences were noted in primary aortic pathology (*P* < 0.001); aortic dissection was more prevalent in the single-branch group (81.7%) than in the double-branch group (41.4%), whereas thoracic aortic aneurysm was more common in the double-branch group (41.4% vs. 9.5%). Overall, aortic dissection accounted for 150/198 (75.8%) cases, which likely explains the relatively young mean age of our cohort. The landing zone distribution also differed significantly (*P* < 0.001), with zone 2 predominantly utilized for single-branch repairs (95.9%) and zone 1 for double-branch repairs (86.2%).

**Table 1:** Baseline demographic and clinical characteristics of patients undergoing Single-Branch versus Double-Branch TEVAR

Variable	Single-Branch (*N* = 169)	Double-Branch (*N* = 29)	*P* value
Age	56.3 ± 14.3	58.3 ± 13.8	0.481
Sex	143 (84.6%)	25 (86.2%)	> 0.99
Smoking history	78 (46.2%)	15 (51.7%)	0.579
Alcohol history	71 (42.0%)	15 (51.7%)	0.33
Coronary heart disease	30 (17.8%)	7 (24.1%)	0.415
Stroke	16 (9.5%)	6 (20.7%)	0.104
Diabetes	13 (7.7%)	1 (3.4%)	0.698
Hyperlipidemia	26 (15.4%)	7 (24.1%)	0.280
Marfan syndrome	2 (1.2%)	0 (0.0%)	> 0.99
COPD	5 (3.0%)	1 (3.4%)	> 0.99
Hypertension	146 (86.4%)	27 (93.1%)	0.543
Renal insufficiency	17 (10.1%)	1 (3.4%)	0.481
Major diseases			< 0.001
AD	138 (81.7%)	12 (41.4%)	
IMH	2 (1.2%)	2 (6.9%)	
PAU	13 (7.7%)	3 (10.3%)	
TAA	16 (9.5%)	12 (41.4%)	
Emergency surgery	21 (12.4%)	1 (3.4%)	0.21
Landing zone			< 0.001
Zone 1	7 (4.1%)	25 (86.2%)	
Zone 2	162 (95.9%)	4 (13.8%)	

COPD, chronic obstructive pulmonary disease; AD, aortic dissection; IMH, intramural hematoma; PAU, penetrating aortic ulcer; TAA, thoracic aortic aneurysm

**Fig. 2:**
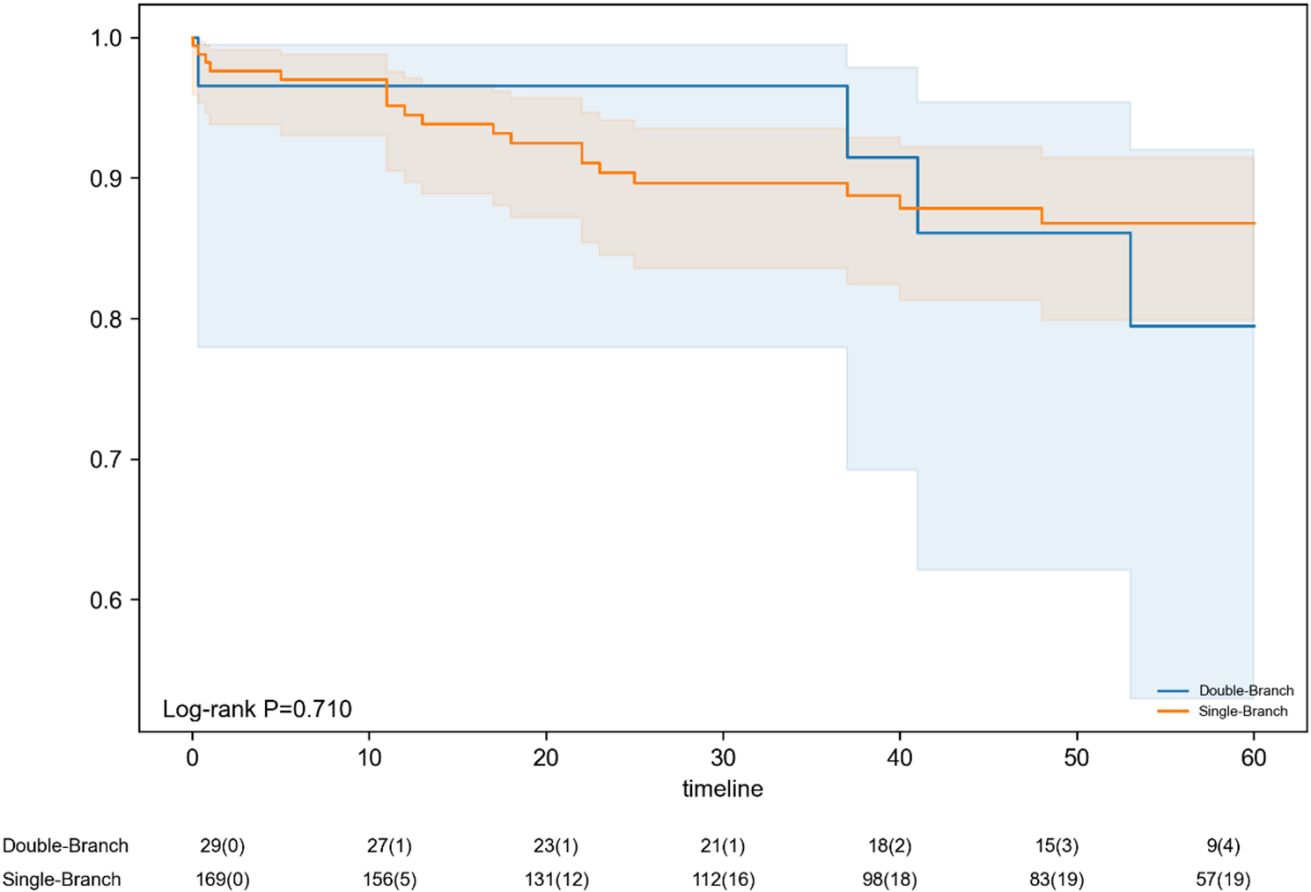
Kaplan-Meier Survival Curves for Mid-term All-Cause Mortality: Single-Branch versus Double-Branch TEVAR. The graph compares all-cause mortality between the single-branch (*n* = 169) and double-branch (*n* = 29) reconstruction groups. The number of patients at risk is shown at the bottom at each time interval. The outcome shown is all-cause mortality; estimates at later time points should be interpreted cautiously because of the small numbers at risk, especially in the double-branch group. The log-rank test was used for comparison (*P* = 0.710). *TEVAR*,* thoracic endovascular aortic repair*

Perioperatively (Table 2), double-branch procedures involved significantly longer mean operative times (261.4 ± 111.7 min vs. 142.9 ± 64.8 min, *P* < 0.001). Despite this, the overall technical success rate was high at 96.0% (190/198) and similar between the groups (single-branch 96.4% vs. double-branch 93.1%, *P* = 0.332). The eight cases recorded as technical failures included type I endoleak (*n* = 4), type III endoleak (*n* = 2), one intraoperative death due to acute myocardial infarction (double-branch group, *n* = 1), and one persistent type II endoleak related to the LSA despite repeat embolization in a hybrid case (*n* = 1). Perioperative all-cause mortality occurred in four patients (2.0% overall) and did not differ significantly between the single-branch (1.8%) and double-branch (3.4%) groups (*P* = 0.472). Among the four perioperative deaths, one was aorta-related due to malperfusion with subsequent multiorgan failure. The remaining three deaths were non-aortic, consisting of one intraoperative death due to acute myocardial infarction, one out-of-hospital sudden death within three weeks after TEVAR, and one intraoperative death during colectomy ten days after TEVAR in a patient with colon cancer. Other perioperative adverse events, including major complications and reintervention rates, were generally low and comparable between the groups, as further detailed in Table 2. The mean follow-up duration was 40.5 months and was similar for both groups (single-branch: 40.5 ± 19.8 months; double-branch: 40.5 ± 20.9 months; *P* = 0.953) (Table 2). Kaplan-Meier survival analysis revealed no statistically significant difference in mid-term all-cause mortality between the two groups (Fig. 2, Log-rank *P* = 0.710).

**Table 2:** Perioperative data and Mid-term outcomes of Single-Branch versus Double-Branch TEVAR

Variable	Single-Branch (*N* = 169)	Double-Branch (*N* = 29)	*P* value
Operation time	142.9 ± 64.8	261.4 ± 111.7	< 0.001
Technical success	163 (96.4%)	27 (93.1%)	0.332
Perioperative all-cause death	3 (1.8%)	1 (3.4%)	0.472
Perioperative aorta-related death	1 (0.6%)	0 (0.0%)	> 0.99
Perioperative access thrombosis	1 (0.6%)	0 (0.0%)	> 0.99
Perioperative access rupture	1 (0.6%)	0 (0.0%)	> 0.99
Perioperative pseudoaneurysm	2 (1.2%)	0 (0.0%)	> 0.99
Perioperative renal insufficiency	2 (1.2%)	0 (0.0%)	> 0.99
Perioperative stroke	2 (1.2%)	1 (3.4%)	0.380
Perioperative MI	1 (0.6%)	1 (3.4%)	0.272
Perioperative lower limb embolism	0 (0.0%)	1 (3.4%)	0.146
Perioperative SMA embolism	0 (0.0%)	1 (3.4%)	0.146
Perioperative renal artery restenosis	0 (0.0%)	0 (0.0%)	-
Perioperative type I endoleak	4 (2.4%)	0 (0.0%)	> 0.99
Perioperative type II endoleak	1 (0.6%)	0 (0.0%)	> 0.99
Perioperative type III endoleak	1 (0.6%)	1 (3.4%)	0.272
Perioperative pSINE	2 (1.2%)	0 (0.0%)	> 0.99
Perioperative dSINE	0 (0.0%)	0 (0.0%)	-
Perioperative RTAD	1 (0.6%)	0 (0.0%)	> 0.99
Perioperative branch stent restenosis requiring reintervention	0 (0.0%)	0 (0.0%)	-
Perioperative adverse event reintervention	2 (1.2%)	2 (6.9%)	0.103
Perioperative aorta-related complication reintervention	2 (1.2%)	1 (3.4%)	0.380
Follow-up time	40.5 ± 19.8	40.5 ± 20.9	0.953
Freedom from reintervention time	36.9 ± 21.6	32.4 ± 23.3	0.233
Follow-up stroke	6 (3.6%)	1 (3.4%)	> 0.99
Follow-up MI	2 (1.2%)	1 (3.4%)	0.380
Follow-up renal insufficiency	3 (1.8%)	0 (0.0%)	> 0.99
Follow-up renal artery restenosis	3 (1.8%)	0 (0.0%)	> 0.99
Follow-up type I endoleak	0 (0.0%)	0 (0.0%)	-
Follow-up type II endoleak	0 (0.0%)	1 (3.4%)	0.146
Follow-up type III endoleak	1 (0.6%)	0 (0.0%)	> 0.99
Follow-up pSINE	3 (1.8%)	0 (0.0%)	> 0.99
Follow-up dSINE	13 (7.7%)	0 (0.0%)	0.222
Follow-up RTAD	3 (1.8%)	0 (0.0%)	> 0.99
Follow-up branch stent restenosis requiring reintervention	3 (1.8%)	2 (6.9%)	0.156
Follow-up adverse event reintervention	4 (2.4%)	0 (0.0%)	> 0.99
Follow-up aorta-related complication reintervention	8 (4.7%)	1 (3.4%)	> 0.99
Follow-up aorta-related complication occurred	16 (9.5%)	1 (3.4%)	0.476
All-cause death during follow-up	19 (11.2%)	4 (13.8%)	0.753
Aorta-related death during follow-up	2 (1.2%)	0 (0.0%)	> 0.99

MI, myocardial infarction; SMA, superior mesenteric artery; pSINE, proximal stent-induced new entry; dSINE, distal stent-induced new entry; RTAD, retrograde type A dissection

To further identify predictors of mortality, multivariable Cox proportional hazards regression analysis (Model 1) was performed, which met the proportional hazards assumption. After adjusting for baseline covariates, older age emerged as an independent predictor of increased mid-term all-cause mortality (HR 1.047 per year, 95% CI 1.012–1.084, *P* = 0.009). In contrast, the type of branch reconstruction (double-branch vs. single-branch) was not an independent predictor of mortality (HR 1.356, 95% CI, 0.420–4.385; *P* = 0.611) (Table 3). Regarding other mid-term outcomes, there were no significant differences between the single-branch and double-branch groups in the rates of aorta-related complications (9.5% vs. 3.4%, *P* = 0.476) or reinterventions for aorta-related complications (4.7% vs. 3.4%, *P* = 1.000), with further details available in Table 2.

**Table 3:** Multivariable Cox proportional hazards regression analysis of Mid-term All-Cause mortality for Single-Branch versus Double-Branch TEVAR (Model 1)

Covariate	HR (exp(coef))	95% CI for HR	*P* value
Procedure Type (Double-branch vs. Single-branch)	1.356	0.420–4.385	0.611
Age (per year increase)	1.047	1.012–1.084	0.009
Sex (Male vs. Female)	1.061	0.360–3.126	0.914
Emergency Surgery (Yes vs. No)	1.907	0.641–5.669	0.246
Diagnosis (Aortic Dissection vs. Other)	1.147	0.419–3.140	0.789

HR, hazard ratio; CI, confidence interval

Because our cohort spans 2015 to 2023, the institutional strategy for supra-aortic reconstruction evolved over time in response to device availability and accumulating operator experience (Supplementary Figures S1–S2). Fenestration-based approaches were used more frequently in the earlier era, whereas physician-modified branched configurations, introduced in 2018, progressively became the predominant single-branch option in later years. Hybrid and chimney techniques were applied selectively, primarily as complementary or bailout strategies in anatomically challenging scenarios. This temporal evolution introduces an important era effect: comparisons across single-branch techniques are observational and may reflect not only differences in device design but also learning-curve effects and changes in patient selection over time. Therefore, our technique-comparison findings should be interpreted as descriptive and hypothesis-generating rather than definitive evidence of superiority.

### Analysis of different reconstruction techniques within Single-Branch TEVAR

An in-depth analysis was conducted on 169 patients who underwent single-branch TEVAR to examine the evolution of technique utilization and comparative outcomes. The application of various reconstruction techniques within this cohort shifted over the study period from 2015 to 2023, as illustrated by the temporal trends in technique utilization, shown in Supplementary Figure S1 and Supplementary Figure S2. Physician-modified branched stent grafts, introduced in 2018, have progressively become the predominant single-branch approach in later years. In contrast, fenestration techniques were more common in the earlier study phase (2016–2017) and saw a relative decline in application thereafter, whereas chimney and hybrid techniques were utilized less frequently throughout the study for single-branch reconstructions. The baseline characteristics of patients categorized by the four primary single-branch techniques (physician-modified branched stent graft, *n* = 72; chimney, *n* = 25; fenestration, *n* = 64; hybrid, *n* = 8) are presented in Table 4. In our cohort, the hybrid approach referred to carotid-to-subclavian bypass followed by intentional LSA coverage during TEVAR; LSA occlusion was achieved predominantly by surgical ligation, while endovascular embolization was used in selected early cases. In addition, seven patients in the single-branch cohort underwent intentional LSA embolization without LSA revascularization, including six cases treated with the Castor single-branched stent graft reconstructing the left common carotid artery (LCCA) and one case treated with physician-modified fenestration of the LCCA. These subgroups were largely comparable across most demographic and clinical variables, although a statistically significant difference was observed in smoking history (*P* = 0.014), with higher rates in the hybrid and chimney groups, and a trend towards difference was noted in the prevalence of coronary heart disease (*P* = 0.053). Perioperative data for these technique subgroups, detailed in Table 5, showed that the operative time differed significantly (*P* < 0.001), with the hybrid approach requiring the longest mean duration. However, technical success rates were consistently high and did not differ significantly across the techniques (*P* = 0.228). Similarly, perioperative mortality and the incidence of most major adverse events showed no significant intergroup differences, with the notable exception of perioperative Type II endoleak, which occurred significantly more frequently in the hybrid technique group (12.5%, 1/8 patients) than in the other groups (0/161 patients, *P* < 0.001).

**Table 4:** Baseline demographic and clinical characteristics by reconstruction technique in Single-Branch TEVAR

Variable	Branched Stent-Graft Technique (*N* = 72)	Chimney Technique (*N* = 25)	Fenestration Technique (*N* = 64)	Hybrid Technique (*N* = 8)	*P* value
Age	56.7 ± 14.8	56.0 ± 13.8	56.5 ± 14.2	52.2 ± 14.4	0.874
Sex	65 (90.3%)	21 (84.0%)	50 (78.1%)	7 (87.5%)	0.272
Smoking history	30 (41.7%)	16 (64.0%)	25 (39.1%)	7 (87.5%)	0.014
Alcohol history	28 (38.9%)	10 (40.0%)	31 (48.4%)	2 (25.0%)	0.500
Coronary heart disease	10 (13.9%)	1 (4.0%)	17 (26.6%)	2 (25.0%)	0.053
Stroke	6 (8.3%)	3 (12.0%)	7 (10.9%)	0 (0.0%)	0.731
Diabetes	5 (6.9%)	3 (12.0%)	5 (7.8%)	0 (0.0%)	0.711
Hyperlipidemia	8 (11.1%)	5 (20.0%)	13 (20.3%)	0 (0.0%)	0.254
Marfan syndrome	0 (0.0%)	0 (0.0%)	2 (3.1%)	0 (0.0%)	0.345
COPD	0 (0.0%)	1 (4.0%)	4 (6.2%)	0 (0.0%)	0.176
Hypertension	60 (83.3%)	23 (92.0%)	58 (90.6%)	5 (62.5%)	0.107
Renal insufficiency	3 (4.2%)	3 (12.0%)	10 (15.6%)	1 (12.5%)	0.164
Major diseases					0.104
AD	62 (86.1%)	20 (80.0%)	51 (79.7%)	5 (62.5%)	
IMH	0 (0.0%)	1 (4.0%)	1 (1.6%)	0 (0.0%)	
PAU	4 (5.6%)	1 (4.0%)	5 (7.8%)	3 (37.5%)	
TAA	6 (8.3%)	3 (12.0%)	7 (10.9%)	0 (0.0%)	
Emergency surgery	10 (13.9%)	1 (4.0%)	9 (14.1%)	1 (12.5%)	0.587
Landing zone					0.128
Zone 1	6 (8.3%)	0 (0.0%)	1 (1.6%)	0 (0.0%)	
Zone 2	66 (91.7%)	25 (100.0%)	63 (98.4%)	8 (100.0%)	

COPD, chronic obstructive pulmonary disease; AD, aortic dissection; IMH, intramural hematoma; PAU, penetrating aortic ulcer; TAA, thoracic aortic aneurysm

**Table 5:** Perioperative data and outcomes by reconstruction technique in Single-Branch TEVAR

Outcome	Branched Stent-Graft Technique (*N* = 72)	Chimney Technique (*N* = 25)	Fenestration Technique (*N* = 64)	Hybrid Technique (*N* = 8)	*P* value
Operation time	143.9 ± 59.0	136.8 ± 75.2	132.2 ± 59.3	239.4 ± 49.0	0.001
Technical success	71 (98.6%)	23 (92.0%)	62 (96.9%)	7 (87.5%)	0.228
Perioperative all-cause death	2 (2.8%)	0 (0.0%)	1 (1.6%)	0 (0.0%)	0.794
Perioperative aorta-related death	1 (1.4%)	0 (0.0%)	0 (0.0%)	0 (0.0%)	0.716
Perioperative access thrombosis	1 (1.4%)	0 (0.0%)	0 (0.0%)	0 (0.0%)	0.716
Perioperative access rupture	0 (0.0%)	0 (0.0%)	1 (1.6%)	0 (0.0%)	0.648
Perioperative pseudoaneurysm	0 (0.0%)	0 (0.0%)	2 (3.1%)	0 (0.0%)	0.345
Perioperative renal insufficiency	0 (0.0%)	1 (4.0%)	1 (1.6%)	0 (0.0%)	0.435
Perioperative stroke	1 (1.4%)	0 (0.0%)	1 (1.6%)	0 (0.0%)	0.919
Perioperative MI	1 (1.4%)	0 (0.0%)	0 (0.0%)	0 (0.0%)	0.716
Perioperative lower limb embolism	0 (0.0%)	0 (0.0%)	0 (0.0%)	0 (0.0%)	-
Perioperative SMA embolism	0 (0.0%)	0 (0.0%)	0 (0.0%)	0 (0.0%)	-
Perioperative renal artery restenosis	0 (0.0%)	0 (0.0%)	0 (0.0%)	0 (0.0%)	-
Perioperative type I endoleak	1 (1.4%)	2 (8.0%)	1 (1.6%)	0 (0.0%)	0.25
Perioperative type II endoleak	0 (0.0%)	0 (0.0%)	0 (0.0%)	1 (12.5%)	< 0.001
Perioperative type III endoleak	0 (0.0%)	0 (0.0%)	1 (1.6%)	0 (0.0%)	0.648
Perioperative pSINE	2 (2.8%)	0 (0.0%)	0 (0.0%)	0 (0.0%)	0.436
Perioperative dSINE	0 (0.0%)	0 (0.0%)	0 (0.0%)	0 (0.0%)	-
Perioperative RTAD	1 (1.4%)	0 (0.0%)	0 (0.0%)	0 (0.0%)	0.716
Perioperative branch stent restenosis requiring reintervention	0 (0.0%)	0 (0.0%)	0 (0.0%)	0 (0.0%)	-
Perioperative adverse event reintervention	0 (0.0%)	0 (0.0%)	2 (3.1%)	0 (0.0%)	0.345
Perioperative aorta-related complication reintervention	1 (1.4%)	0 (0.0%)	1 (1.6%)	0 (0.0%)	0.919

MI, myocardial infarction; pSINE, proximal stent-induced new entry; RTAD, retrograde type A dissection

Regarding mid-term outcomes for these single-branch techniques, Kaplan-Meier survival analysis demonstrated no overall significant difference in all-cause mortality among the four main reconstruction approaches (Supplementary Figure S3, Log-rank P = 0.379). An attempt to further elucidate predictors of mortality using multivariable Cox regression analysis (Model 2, Table 6), comparing Branched Stent-Graft and fenestration techniques against the chimney technique (excluding the hybrid group due to small numbers and zero mortality events), was limited as the proportional hazards assumption was not met for the ‘Branched Stent-Graft’ variable (PH test *P* = 0.031). While this model did not identify technique type as an independent predictor of mortality, coronary heart disease showed a non-significant trend towards increased risk (HR 2.330, *P* = 0.118). Given these limitations of the Cox model, Restricted Mean Survival Time (RMST) analysis was employed as a more robust alternative for comparing survival. The RMST analysis confirmed no statistically significant differences in the mean survival time for all-cause mortality among the four techniques at 12, 24, 36, and 60 months (all pairwise *P* > 0.050). Detailed RMST values at the 60-month time point, with comparisons against the chimney technique, are provided in Table 7 and are visualized in Supplementary Figure S4. The other follow-up outcomes for the single-branch technique subgroups are shown in Table 8. The mean follow-up duration varied significantly across the groups (*P* < 0.001). During this period, there were no statistically significant differences observed in the rates of aorta-related complications or branch stent restenosis requiring reintervention across technique groups. Restenosis requiring reintervention was rare, occurring in 1.8% (3/169) of single-branch patients overall. Furthermore, Kaplan-Meier analysis for freedom from any aortic- or branch-related reintervention showed no significant difference among the technique groups (Log-rank *P* = 0.330).

**Table 6:** Multivariable Cox proportional hazards regression analysis of Mid-term All-Cause mortality by reconstruction technique in Single-Branch TEVAR (Model 2)

Covariate	HR (exp(coef))	95% CI for HR	*P*-value
Reconstruction Technique (Reference: Chimney)			
Branched Stent-Graft (vs. Chimney)	1.909	0.388–9.382	0.426
Fenestration (vs. Chimney)	1.128	0.220–5.791	0.885
Age (per year increase)	1.029	0.987–1.073	0.176
Sex (Male vs. Female)	0.621	0.187–2.065	0.437
Smoking History (Yes vs. No)	1.671	0.594–4.702	0.331
Coronary Heart Disease (Yes vs. No)	2.33	0.807–6.725	0.118
Hypertension (Yes vs. No)	0.622	0.171–2.267	0.472

HR, hazard ratio; CI, confidence interval

**Table 7:** Restricted mean survival time (RMST) comparison for Mid-term All-Cause mortality by reconstruction technique in Single-Branch TEVAR (at 60 Months)

Technique Subgroup	*N*	RMST at 60 months (months) [95% CI]	RMST Difference vs. Chimney (months)	*P*-value (vs. Chimney)
Chimney Technique	25	57.13 [54.17–60.10]	Reference	-
Branched Stent-Graft Technique	72	52.18 [50.51–53.85]	−4.95	0.309
Fenestration Technique	64	55.43 [53.61–57.26]	−1.7	0.789
Hybrid Technique	8	60.00 [54.63–65.37]	2.87	0.368

RMST, restricted mean survival time; CI, confidence interval

**Table 8:** Follow-up outcomes by reconstruction technique in Single-Branch TEVAR

Outcome	Branched Stent-Graft Technique (*N* = 72)	Chimney Technique (*N* = 25)	Fenestration Technique (*N* = 64)	Hybrid Technique (*N* = 8)	*P* value
Follow-up time	30.4 ± 19.3	42.0 ± 18.1	49.7 ± 16.5	52.9 ± 7.8	< 0.001
Freedom from reintervention time	26.5 ± 19.6	39.9 ± 19.6	46.0 ± 20.0	47.8 ± 16.4	< 0.001
Follow-up stroke	1 (1.4%)	1 (4.0%)	4 (6.2%)	0 (0.0%)	0.448
Follow-up MI	1 (1.4%)	1 (4.0%)	0 (0.0%)	0 (0.0%)	0.46
Follow-up renal insufficiency	2 (2.8%)	0 (0.0%)	1 (1.6%)	0 (0.0%)	0.794
Follow-up renal artery restenosis	3 (4.2%)	0 (0.0%)	0 (0.0%)	0 (0.0%)	0.249
Follow-up type I endoleak	0 (0.0%)	0 (0.0%)	0 (0.0%)	0 (0.0%)	-
Follow-up type II endoleak	0 (0.0%)	0 (0.0%)	0 (0.0%)	0 (0.0%)	-
Follow-up type III endoleak	0 (0.0%)	1 (4.0%)	0 (0.0%)	0 (0.0%)	0.122
Follow-up pSINE	2 (2.8%)	0 (0.0%)	0 (0.0%)	1 (12.5%)	0.063
Follow-up dSINE	6 (8.3%)	0 (0.0%)	7 (10.9%)	0 (0.0%)	0.291
Follow-up RTAD	2 (2.8%)	0 (0.0%)	0 (0.0%)	1 (12.5%)	0.063
Follow-up branch stent restenosis requiring reintervention	3 (4.2%)	0 (0.0%)	0 (0.0%)	0 (0.0%)	0.249
Follow-up adverse event reintervention	3 (4.2%)	0 (0.0%)	1 (1.6%)	0 (0.0%)	0.575
Follow-up aorta-related complication reintervention	4 (5.6%)	1 (4.0%)	2 (3.1%)	1 (12.5%)	0.665
Follow-up aorta-related complication occurred	7 (9.7%)	1 (4.0%)	7 (10.9%)	1 (12.5%)	0.771
All-cause death during follow-up	10 (13.9%)	2 (8.0%)	7 (10.9%)	0 (0.0%)	0.618
Aorta-related death during follow-up	1 (1.4%)	0 (0.0%)	1 (1.6%)	0 (0.0%)	0.919

MI, myocardial infarction; pSINE, proximal stent-induced new entry; dSINE, distal stent-induced new entry; RTAD, retrograde type A dissection

### Exploratory analysis of age and key clinical outcomes

The influence of age on key clinical outcomes was further explored using regression analyses, treating age as a continuous variable (per year) without a prespecified cutoff, with age 57 years used as the reference point in the Restricted Cubic Spline (RCS) models. Regarding follow-up all-cause mortality, RCS regression with three knots identified age as a significant overall predictor (Overall *P* = 0.008). This analysis demonstrated that the relationship between age and mortality risk was primarily linear (P for nonlinearity = 0.107), as illustrated in Supplementary Figure S5. For the association between age and aorta-related complications defined as endoleak, SINE, or RTAD, a 3-knot RCS model demonstrated that this relationship was also predominantly linear (P for nonlinearity = 0.623), with the overall P-value for the effect of age being 0.086 as shown in Supplementary Figure S6, indicating an inverse trend. Consistent with this pattern, univariable logistic regression estimated an OR of 0.957 per year increase (95% CI 0.923–0.993; *P* = 0.020). Finally, branch-specific outcomes were descriptively assessed. During follow-up, branch stent restenosis requiring reintervention occurred in 3 of 169 single-branch patients (1.8%). Given the low event count, regression modeling for this endpoint was not further pursued.

## Discussion

In this single-center cohort of 198 complex aortic arch repairs performed between 2015 and 2023, we demonstrated the feasibility of advanced endovascular techniques and found a signal of an inverse association between older age and aorta-related complications, a finding that is most plausibly explained by selection bias. Our high technical success rate of 96.0% and favorable mid-term mortality rates are consistent with findings from other specialized centers and systematic reviews [12, 13]. Regarding survival, we acknowledge that the Kaplan–Meier curves suggest an approximately 5-year all-cause survival near 80% in some subgroups. However, this should not be interpreted as evidence of device inferiority, but rather in the context of our endpoint definition and patient population. Specifically, the primary endpoint of all-cause mortality captures non-aortic deaths in a cohort characterized by urgent AAS presentations and a substantial baseline comorbidity burden. Furthermore, statistical precision at late time points is limited by the small numbers at risk, particularly in the double-branch subgroup (*n* = 29). Crucially, aorta-related mortality remained low in our cohort (1.5% overall, 3/198), underscoring acceptable device performance and durability despite the observed all-cause survival estimates.

The decision to undertake double-branch repair remains technically demanding, a fact underscored in our cohort by markedly longer operative times and the well-documented technical and anatomical challenges associated with the procedure. For instance, reports on double-branched endografts highlight significant rates of perioperative stroke, and studies on anatomic suitability have shown that a large proportion of patients may not be eligible for currently available devices [14, 15]. In our cohort, the predominance of zone 1 sealing in the double-branch group reflects deliberate case selection: double-branch reconstruction was reserved for patients in whom preoperative centerline CTA confirmed an adequate measurable proximal seal length at zone 1, whereas anatomies with an absent or extremely short zone 1 were preferentially managed using alternative strategies. Against this background, we did not detect a statistically significant difference in mid-term survival compared with single-branch strategies (Log-rank *P* = 0.710). These results should be interpreted with caution, as the small sample size of the double-branch cohort (*n* = 29) limited the statistical power to detect clinically meaningful differences, and the comparison should not be interpreted as evidence of equivalence. Although direct comparisons are challenging, our findings are generally aligned with broader evidence, such as the international analysis of the RELAY™ Branched device, which also found comparable short- and mid-term survival between its single- and double-branch configurations, although clinical outcomes with the single-branch device appeared to be more favorable [16].

Within single-branch reconstructions, our analysis underscores the importance of a tailored strategy and a diverse therapeutic armamentarium, as no single modality proved universally superior in terms of mid-term survival, a finding robustly confirmed by RMST analysis. Instead, the optimal technique selection depends on individual anatomical considerations and operator expertise. Consistent with a meta-analysis identifying a higher rate of postoperative endoleaks with the chimney technique, our cohort also observed perioperative type I endoleak in the chimney subgroup (2 of 25 patients (8.0%)), although this trend was not statistically significant (*P* = 0.250) [17]. Notably, these initial challenges appeared to resolve during early follow-up, as both patients showed complete resolution on their 1-month CTA without requiring reintervention, a pattern consistent with spontaneous sealing, possibly related to gutter thrombosis; furthermore, no new Type Ia endoleaks were detected in any single-branch group during the entire follow-up period. The trend towards increased use of branched stent grafts in our center is supported by literature demonstrating excellent outcomes such as high branch patency and effective aortic remodeling [18, 19]. Meanwhile, hybrid techniques, which were associated with longer operative times in our cohort, remain an indispensable option, with studies showing reasonable mid-term outcomes in real-world practice, particularly when used as a complementary or bailout strategy in selected patients, and demonstrating excellent long-term durability of surgical bypasses [20, 21].

A key finding of our study was the multifaceted and seemingly paradoxical role of patient age. Our Cox regression analysis robustly identified older age as an independent predictor of all-cause mortality, a finding strongly supported by large-scale studies, such as that by Scali et al., who identified age > 70 years as a powerful predictor of 1-year mortality after TEVAR [22, 23]. Accordingly, “older age” in this study refers to increasing age on a continuous scale rather than a dichotomous threshold; for interpretability, the adjusted hazard associated with a 10-year age increase corresponds to approximately a 58% higher hazard of all-cause mortality (1.047^10 ≈ 1.58) in Model 1. However, we observed an inverse association between older age and the occurrence of aorta-related complications, including endoleak, stent-induced new entry, and retrograde type A dissection. The primary driver for this paradoxical finding is almost certainly selection bias, including a healthy survivor effect, whereby clinicians tend to reserve complex interventions for the fittest elderly candidates [24, 25]. In our clinical practice, we are understandably cautious when considering elderly individuals for complex arch TEVAR, likely selecting only those with the most favorable anatomy, such as less tortuosity, larger landing zones, minimal calcification, and better physiological reserves for such demanding procedures, thus creating an artificially “healthier” elderly cohort [26]. The observed linear nature of this association (P for nonlinearity = 0.623) further supports this interpretation. This suggests that, rather than applying a strict age cutoff, surgeons increasingly apply more stringent selection criteria with advancing age. This strong reliance on subjective assessment underscores a critical gap in current practice: the lack of robust and validated risk-stratification models specifically designed for elderly patients undergoing complex arch TEVAR. This finding highlights the urgent need for geriatric-specific risk scores to guide patient selection in this evolving era of arch repair.

Although selection bias is the most plausible explanation, our observational data cannot disentangle anatomical selection from potential age-related changes in aortic wall properties. Future multicenter studies incorporating standardized anatomical measurements and biomechanical assessments may help clarify these mechanisms [27–29]. Branch-related restenosis events requiring reintervention were rare in our cohort. Given the low event count, regression modeling for this endpoint was not pursued, and the findings should be interpreted descriptively. Prior studies suggest that branch patency may be influenced predominantly by local technical and hemodynamic factors, including sealing zone diameter and device characteristics [30].

Our study had several important limitations. The retrospective design not only introduces the critical selection bias discussed above but also introduces potential information bias. The study period witnessed a significant evolution in the techniques. This era effect is a major confounder for technique-level comparisons and may reflect both technological evolution and the institutional learning curve [31, 32]. Finally, the small sample size, particularly for the double-branch and certain single-branch subgroups, limits our ability to draw definitive conclusions regarding smaller effect sizes. In addition, imaging follow-up was not uniformly available for all patients, and telephone follow-up was used primarily to ascertain survival status and major clinical events when scheduled imaging was unavailable. Imaging-defined endpoints were derived strictly from available computed tomography angiography, which may have led to under-ascertainment of asymptomatic complications in patients lost to imaging follow-up.

## Conclusion

In conclusion, complex arch TEVAR with branch reconstruction is technically feasible and yields acceptable mid-term outcomes in carefully selected patients. While no statistically significant differences in mid-term survival were observed between single-branch and double-branch strategies in our cohort, different single-branch techniques showed broadly comparable mid-term outcomes, reinforcing the need for an individualized, anatomy-driven approach. Most importantly, the observed inverse association between age and aorta-related complications, alongside the expected association between age and higher all-cause mortality, underscores the critical role of patient selection in advanced endovascular procedures and should be interpreted as hypothesis-generating given the retrospective design and potential selection bias. Future multicenter studies with standardized anatomical assessment and imaging follow-up are needed to clarify the interplay between patient selection, aortic wall properties, and long-term device-related outcomes in the elderly.

## Supplementary Information

Supplementary Material 1

## Data Availability

The datasets generated and analyzed during the current study are not publicly available due to patient privacy regulations but are available from the corresponding author on reasonable request.
