# Supplementary material for: Anatomy-driven strategies and mid-term outcomes of branched endovascular repair of the aortic arch: a single-center cohort study
Source: J Cardiothorac Surg. 2026 Feb 9;21:130. doi: 10.1186/s13019-026-03884-6 (PMC12983832; doi:10.1186/s13019-026-03884-6)

**Supplementary Figure S1. Annual Trends in the Utilization of Branched TEVAR Techniques for Aortic Arch Lesions (N=198).** Bar chart showing the annual number of all branched TEVAR procedures performed at our center from 2015 to 2023. Different colors represent different reconstruction techniques applied across the entire cohort. *TEVAR, thoracic endovascular aortic repair.*


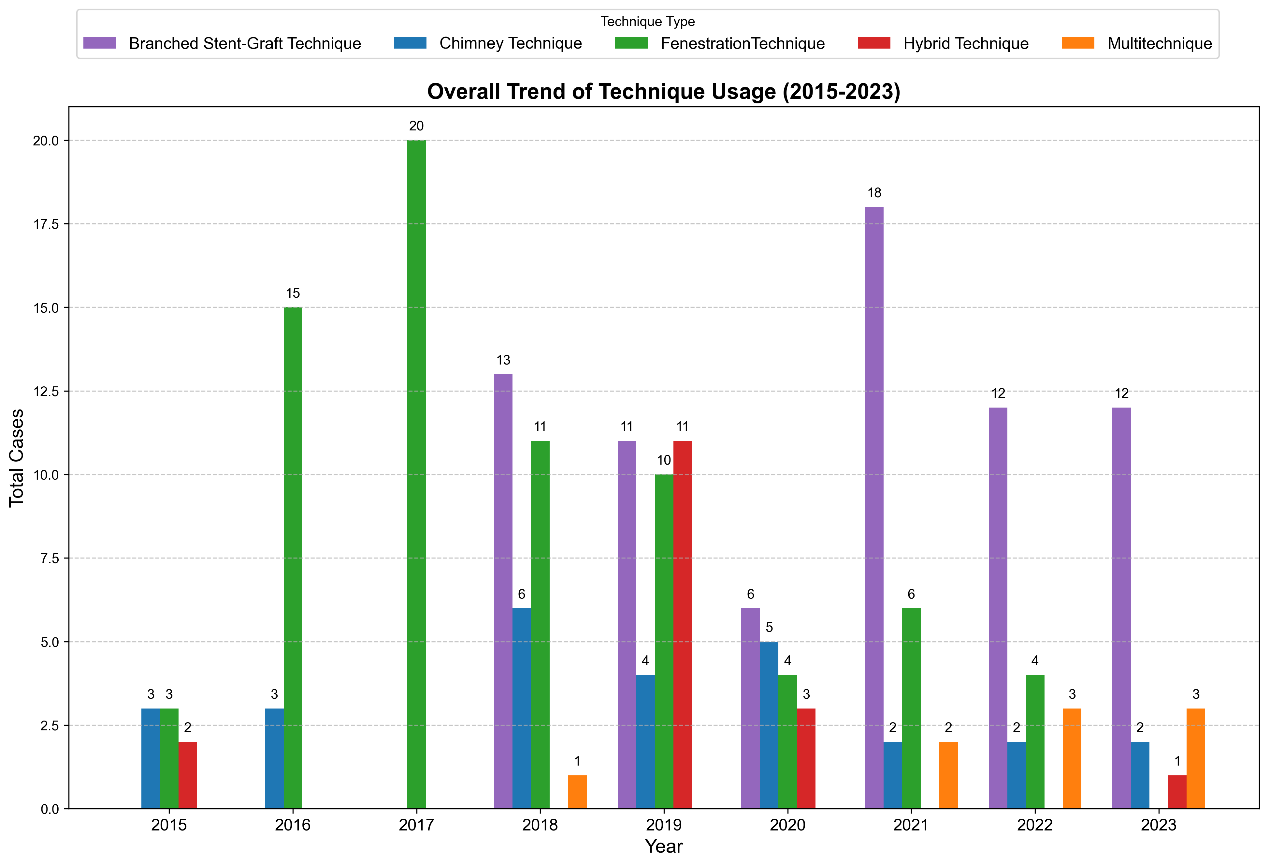


**Supplementary Figure S2. Annual Trends in the Proportional Utilization of Different Single-Branch Reconstruction Techniques (N=169).** Stacked area chart illustrating the annual proportional use of the four main single-branch reconstruction techniques: physician-modified branched stent-graft, chimney, fenestration, and hybrid. *TEVAR, thoracic endovascular aortic repair.*


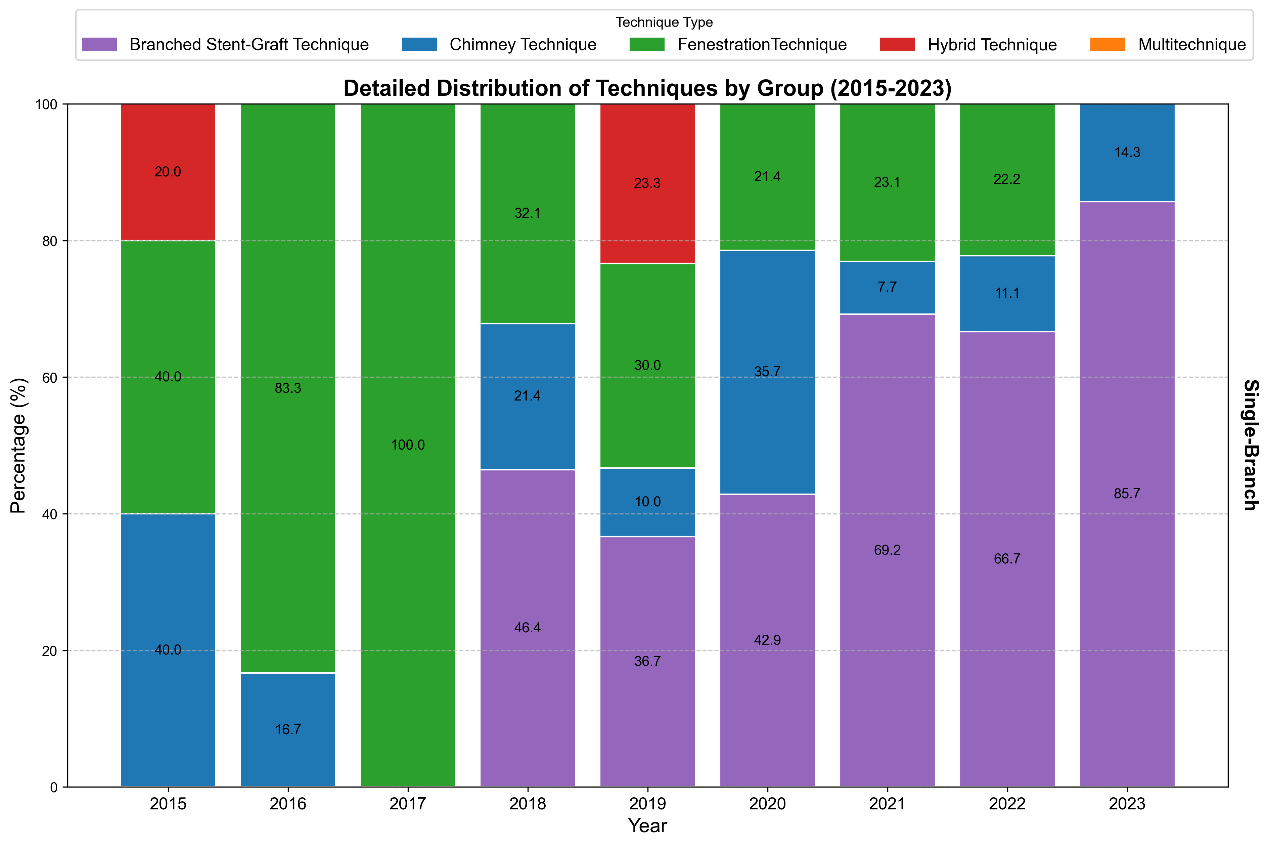


**Supplementary Figure S3. Kaplan-Meier Survival Curves for Mid-term All-Cause Mortality by Single-Branch Reconstruction Technique.** The graph compares all-cause mortality among the four main single-branch reconstruction techniques: physician-modified branched stent-graft (n=72), chimney (n=25), fenestration (n=64), and hybrid (n=8). The number of patients at risk is shown at the bottom at each time interval. The outcome shown is all-cause mortality; estimates at later time points should be interpreted cautiously because of the small numbers at risk, especially in the hybrid and chimney subgroups. The log-rank test was used for comparison (P = 0.379). *TEVAR, thoracic endovascular aortic repair.*


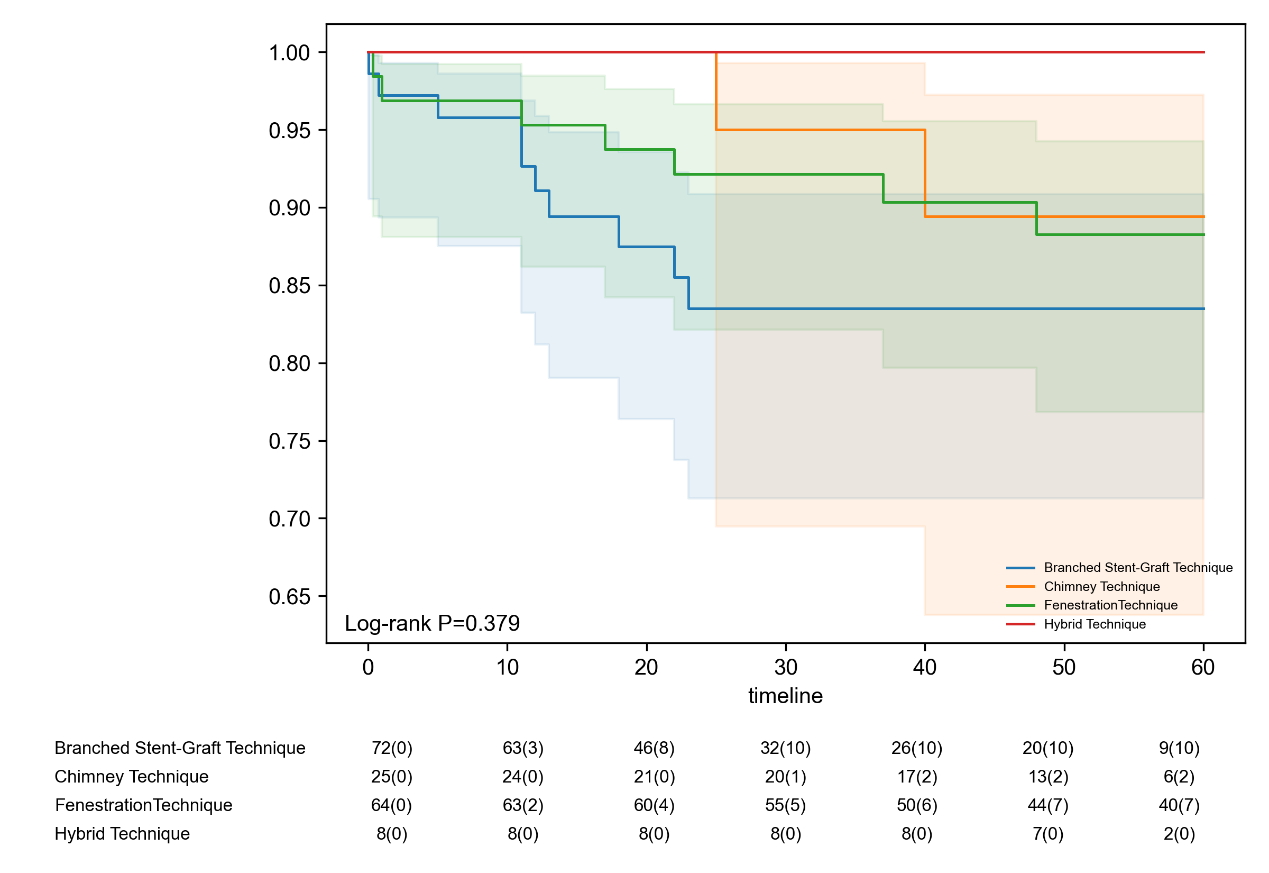


**Supplementary Figure S4. Restricted Mean Survival Time (RMST) Comparison for Mid-term All-Cause Mortality by Single-Branch Reconstruction Technique.** Forest plot visually represents the comparison of the Restricted Mean Survival Time (RMST) among the four single-branch reconstruction techniques at the 60-month time horizon. The plot shows the RMST difference and 95% confidence intervals for each technique compared to the chimney technique as the reference group. *TEVAR, thoracic endovascular aortic repair.*


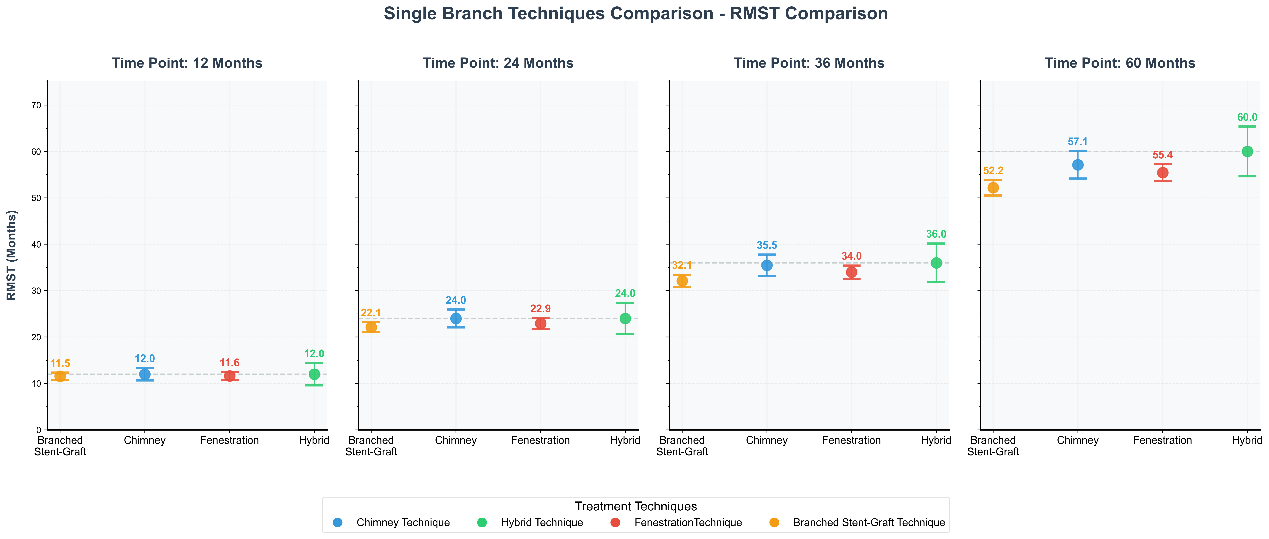


**Supplementary Figure S5. Restricted Cubic Spline (RCS) Curve for the Association between Age and Follow-up All-Cause Mortality.** The curve illustrates the predicted odds ratio of all-cause mortality as a continuous function of patient age, based on a logistic regression model with restricted cubic splines (3 knots, reference age: 57 years). The shaded area represents the 95% confidence interval. P-value for the overall association is 0.008, and the P-value for non-linearity is 0.107. *TEVAR, thoracic endovascular aortic repair.*


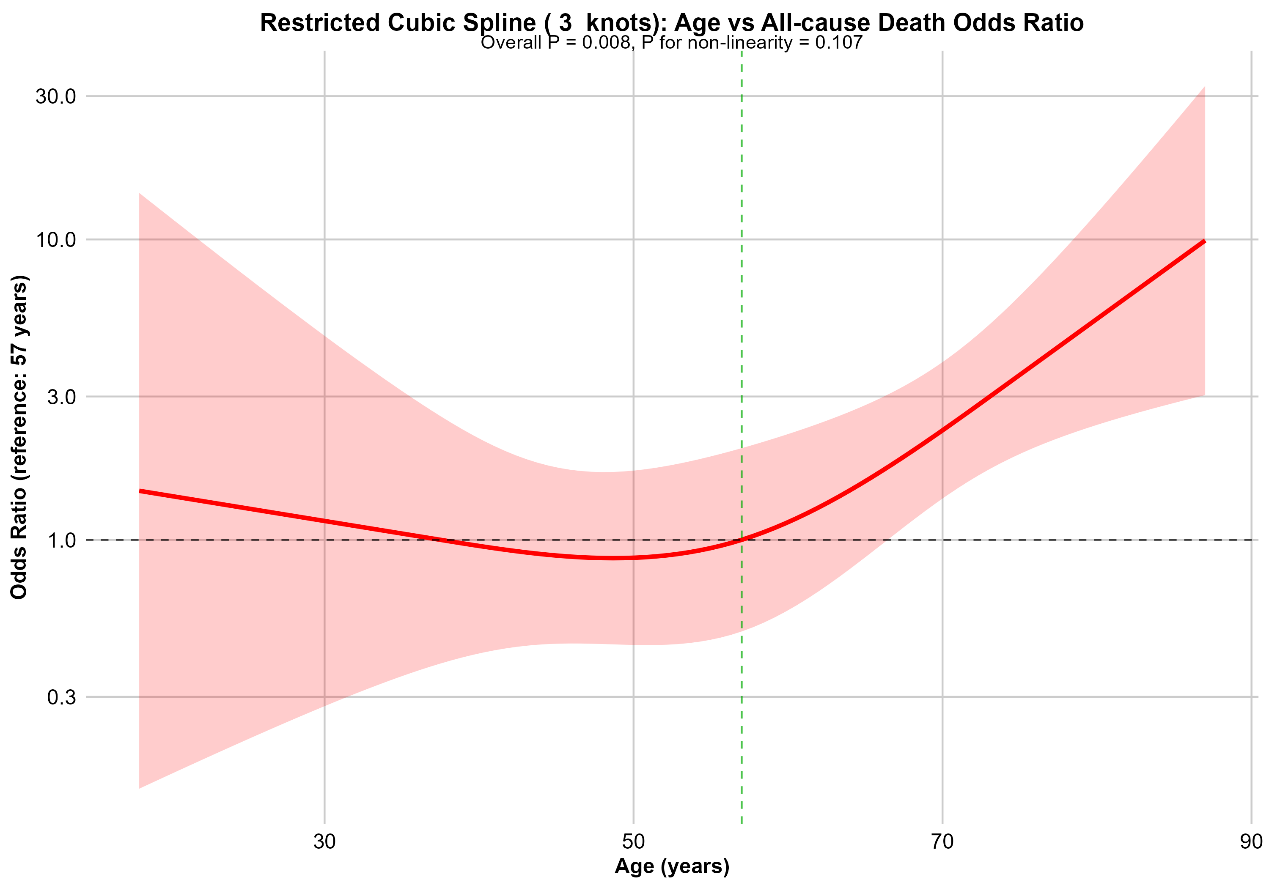


**Supplementary Figure S6. Restricted Cubic Spline (RCS) Curve for the Association between Age and Follow-up Aorta-related Complications.** The curve illustrates the predicted odds ratio of aorta-related complications (defined as endoleak, SINE, or RTAD) as a continuous function of patient age, based on a logistic regression model with restricted cubic splines (3 knots, reference age: 57 years). The shaded area represents the 95% confidence interval. P-value for the overall association is 0.086, and the P-value for non-linearity is 0.623. *TEVAR, thoracic endovascular aortic repair.* *SINE: Stent-Induced New Entry; RTAD: Retrograde Type A Dissection.*


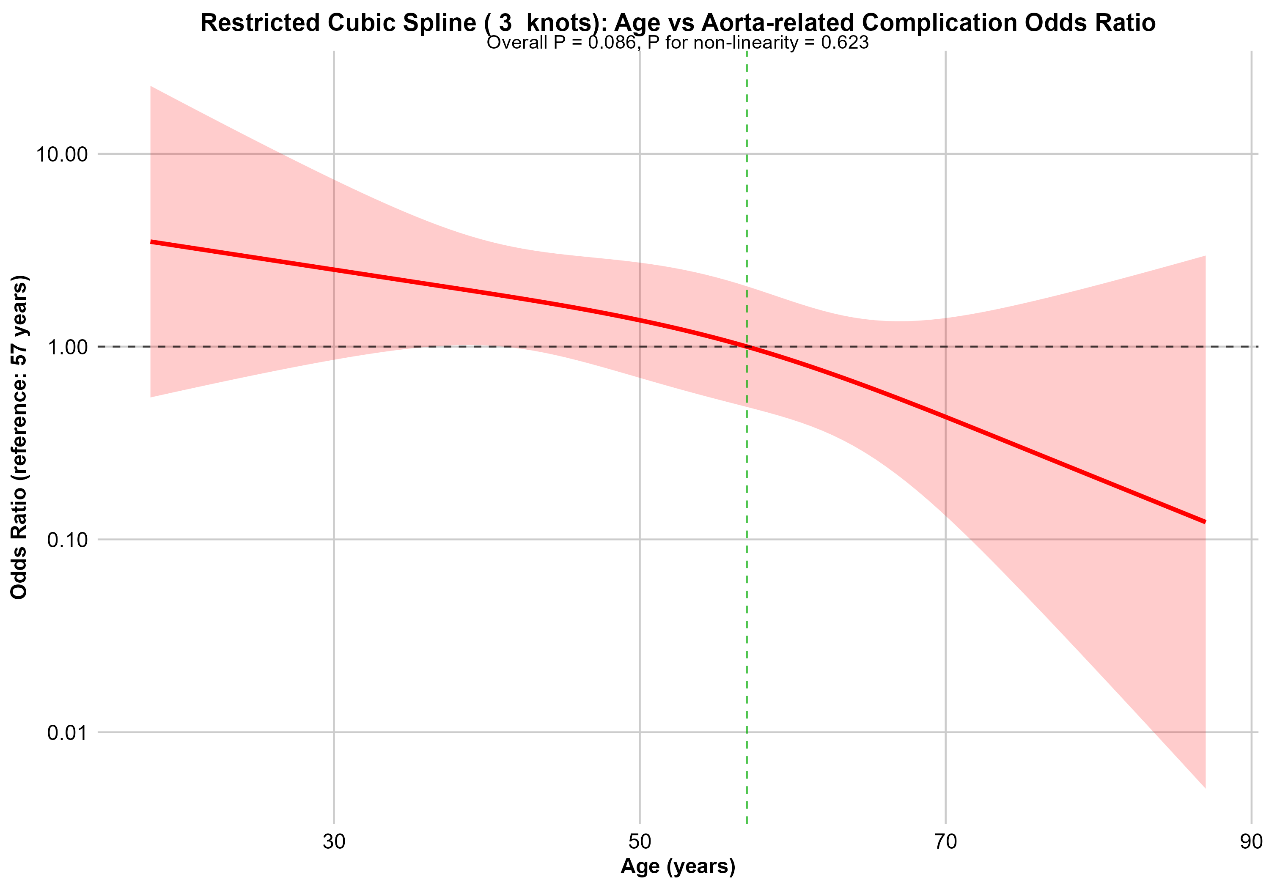

Supplement: Supplementary file 1 — Supplementary Material 1 [file 13019_2026_3884_MOESM1_ESM.docx]
